# Trajectories of physical activity during COVID-19: A longitudinal analysis of UK young adults

**DOI:** 10.1371/journal.pone.0289416

**Published:** 2023-08-09

**Authors:** William John Robert Thorpe, Leslie Morrison Gutman

**Affiliations:** Department of Clinical, Educational and Health Psychology, University College London, London, United Kingdom; Brunel University London, UNITED KINGDOM

## Abstract

Research has demonstrated a disproportionate reduction in the moderate-vigorous physical activity (MVPA) of young UK adults during the initial months of COVID-19. However, previous research has not examined the trajectory of MVPA for this demographic over subsequent phases of the pandemic. The present study investigated the trajectory of MVPA from April 2020 to January 2021 in young UK adults. Data were drawn from 18-29-year-old participants of the Understanding Society COVID-19 Survey (212 males, 542 females). Weekly MVPA was self-reported at three time points (April and September 2020; January 2021) using the International Physical Activity Questionnaire. After controlling for significant covariates, growth curve modelling revealed no variation in weekly MVPA, which remained higher than the UK Physical Activity Guidelines. Female gender; Asian, Black and Mixed ethnicity; lower income,; living with a partner; and no access to a private garden or other outdoor space were associated with lower MVPA in April 2020. Gender, however, was a significant moderator of the trajectory. Males’ MVPA increased between April 2020 to August 2020, followed by a sharp decline; whereas females showed a steady rate of decline from April 2020 to January 2021. Despite the recurrent lockdowns, this study shows that young UK adults, on average, continued to engage in MVPA, above the recommendated amount. Nevertheless, significant variation associated with gender, ethnicity and income highlights the importance of providing accessible spaces for young adults to exercise, especially those with limited access to private gardens.

## Introduction

Moderate-vigorous physical activity (MVPA) promotes positive physical and mental health such as reducing the incidence of depression, anxiety, and heart disease [1], with 75–150 minutes per week recommended for optimal health [2], Research examining trends during COVID-19 documents declining MVPA, falling below the recommended baseline during the first UK lockdown, then showing moderate improvement thereafter [3]. Young UK adults (18–29 years), in particular, reported a disproportionate decline in MVPA between late March and August 2020 [4]. However, no studies to date have investigated the trajectory of MVPA and its associated covariates over the three UK lockdowns for this demographic group, which may usefully direct immediate interventions towards sub-populations at higher-risk of pandemic-related MVPA decline.

A number of demographic factors have been shown to be associated with MVPA for young adults in the UK during the COVID-19 pandemic. Young UK women reported a markedly higher average decrease compared to men between March and June 2020 [5]. However, Bu and colleagues [4] report no significant difference in MVPA rates between UK males and females from late March until early August 2020, warranting further longitudinal examination to clarify the association with gender and exercise engagement over the course of the pandemic. Moreover, lower household income and living alone were associated with lower MVPA for UK adults during the first lockdown [4, 6]. Similarly pronounced declines were evident in certain ethnic categories, with Black, Asian, and Minority Ethnic (BAME) UK residents reporting greater reductions in physical activity compared to White participants from March-June 2020 [7]. Finally, reduced garden space was correlated with lower MVPA rates in UK adults ages 20–70+ during initial restrictions [8].

In addition to demographic indicators, research highlights the importance of health-related covariates for MVPA during the early pandemic. Individuals with a psychiatric or physical health condition, high levels of emotional disturbance, and cigarette use reported lower MVPA outcomes during the first lockdown [4, 9, 10]. Busse and colleagues suggest these findings reflect a clustering effect commonly observed within epidemiological research, wherein health-related behaviours and conditions appear to co-occur [9]. As pandemic restrictions are strongly associated with the deterioration of physical, mental, and behavioural health outcomes [11–13], a re-examination of these health-related factors on longitudinal MVPA outcomes is essential.

### Current study

The present study examines the trajectory of MVPA from April 2020 to January 2021 for young adults (ages 18 to 29) and investigates differences in the mean level and rate of change for key demographic and health-related variables. Data were drawn from the Understanding Society COVID-19 Study. Based on previous research conducted during and shortly after the first lockdown, we expect; (a) MVPA outcomes to model restrictive measures, improving during periods of eased restrictions and declining in line with the three national lockdowns [3]; (b) worse MVPA outcomes for women, ethnic minorities, individuals with a lower income, individuals living alone, individuals with a pre-existing physical or mental health diagnoses, individuals who report greater cigarette use, and individuals with limited access to outdoor space [4, 5, 8, 10].

## Methodology

### Participants and data source

Secondary data were derived from the Understanding Society COVID-19 Survey, a nationally representative, longitudinal panel study of UK households participating in the Understanding Society UK Household Longitudinal Study (UKHLS). The Understanding Society COVID-19 Survey data are available through the UK Data Service (https://ukdataservice.ac.uk/). The data can be downloaded, ordered or analysed online by registering and accepting their End User Licence. The overall method for gaining consent for participation is oral (see https://www.understandingsociety.ac.uk/documentation/mainstage/consents, for more information about consent). Questionnaires were sent monthly between April 2020 and June 2020, bi-monthly between September 2020 and March 2021, and finally in September 2021. Questionnaires for waves 1 to 4 were sent to participants who had responded to waves 8 or 9 of the UKHLS. Questionnaires for waves 5 to 8 were sent to participants who had recorded at least one partially complete response for any of the initial four COVID-19 survey waves. Wave 9 questionnaires were distributed to participants invited to wave 8. For each completed survey, participants were awarded £2. A final £10 payment was awarded for completion of the wave 9 survey, marking the end of the study. Ethical approval was granted by the University of Essex Ethics Committee for the primary data collection (ETH1920-1271). The present secondary data analysis was exempt from ethical approval.

The current study examined data from 18-29-year-old-adults from Waves 1 (April 2020), 5 (September 2020), and 7 (January 2021) as these were the only waves that assessed MVPA. Participants who did not provide responses for covariates collected at Wave 1 and did not provide two or more responses for the MVPA outcome were excluded from the final analytic sample due to the requirements of growth curve modelling (GCM).

### Measures

Table 1 presents the wave of measurement, ranges, means and standard deviations of measures used for the MVPA model.

**Table 1:** Characteristics of measures for the model predicting MVPA.

Measure	Wave of measurement	Min.	Max.	Mean	^a^SD
Age	1	18	29	24.33	3.38
Household Income	1	1	15	7.31	4.13
Total Household Composition	1	0	8	2.37	1.51
Living with Partner	1	0	1	.40	.49
No garden	2	0	1	.09	.29
Private garden	2	0	1	.79	.41
Shared Garden	2	0	1	.05	.23
Balcony, Rooftop Garden or Terrace	2	0	1	.04	.20
Other Outdoor space	2	0	1	.05	.21
Mental Health Condition	1	0	1	.06	.24
Physical Health Condition	1	0	5	.27	.55
Cigarette Use	1	0	5	2.05	1.21
MVPA Wave 1	1	0	16.00	3.43	3.44
MVPA Wave 2	5	0	16.00	3.31	4.00
MVPA Wave 3	7	0	16.00	2.21	3.59

^a^SD = Standard deviation

*Gender* was coded as a dichotomous variable where the value “1” was assigned to male participants and “0” to female participants.

*Ethnicity* was dummy coded into five ethnic categories including, White, Mixed, Asian, Black, and Other.

*Household Income* was coded as a 15-interval categorical variable where each interval represented a £5000 increase in annual income (e.g., 1 = “£0–£5000”; 15 = “£70,001 and greater”).

*Total Household Composition* was a count of five variables indicating the number of household members in the age ranges of: “0–4”; “5–15”; “16–18”; “19–69”; “70 or older”. A higher score indicated a greater number of household members.

*Living With Partner* was a dichotomous variable drawn from the question “are you currently living with a partner?” where “yes” was assigned the value “1” and “no” the value “0”.

*Access to Outdoor Space* was measured using five dichotomous variables indicating whether the participant has access to: a private garden; shared garden; balcony, rooftop garden or terrace; other outdoor space; or no access. For each variable, “yes” responses were coded with the value “1” and “no” responses with the value “0”.

*Mental Health Condition* was a single question asked whether the participant had a pre-existing emotional, nervous, or psychiatric problem. “yes” responses were coded with the value “1” and “no” responses with the value “0”.

*Physical Health Condition* was a count of 20 items asking whether participants had a specific pre-existing physical health condition. A range of conditions was covered including asthma, diabetes, cancer or malignancy, epilepsy, H.I.V. and other medical conditions.

*Cigarette Use* was a continuous variable derived from the question “Approximately how many cigarettes a day do you usually smoke, including those you roll yourself?” Responses were coded as: 0 = “none”; 1 = “1–5”; 2 = “6–10”; 3 = “11–15”; 4 = “16–20”, 5 = “20 or more”.

*Moderate-Vigorous Physical Activity (MVPA)* was a continuous variable represented in waves 1, 5, and 7, indicating the total hours spent engaging in moderate and vigorous physical activities during the week prior to the survey completion. Measured using the International Physical Activity Questionnaire, moderate activities denoted those that “make you breathe *somewhat* harder than normal”. Vigorous activities denoted those that “make you breathe *much* harder than normal”. For each intensity, a total time variable was calculated by multiplying participants’ estimates for the hours spent engaging in PA on a typical day by their estimate for the number of days spent engaging in PA over the previous 7 days. The total variables for moderate and vigorous PA were then summed to calculate the combined MVPA total. Totalled estimates of 16 hours or more (*n* = 237) for each intensity were removed from the data set to account for outliers and overestimation [14]. Use of the MVPA weekly total permitted comparison against the 75–150-minute baseline recommended by the UK Physical Activity Guidelines to attain mental and physical health benefits [2].

### Statistical analysis

GCM was utilised to explore the trajectory of MVPA (April 2020-January 2021). GCM was selected for the present data set as it accounts for missing data through maximum likelihood estimation and does not require equal time interval spacing across measurement waves [15]. All statistical analyses were conducted using SPSS 27.

To assess the linear rate of change across each assessment wave, a time variable was devised reflecting the number of months passed since wave 1: Wave 1 = 0, Wave 5 = 5, and Wave 7 = 9. To assess the variable rate of change across each assessment wave, a quadratic time variable was devised by squaring the time variable at each wave. The model was constructed by incorporating the linear and quadratic time components, and significant covariates. Interaction terms between the covariates and time components were tested to assess their association with the rate of change in MVPA outcomes.

## Results

The final analytic sample included 754 participants: 75.7% were White, 4.1% Mixed Ethnicity, 10.6% Asian, 1.3% Black, and 0.3% Other. Females comprised 72.3% of the sample. One-way ANOVA was used to assess differences between the analytic sample (*n* = 757) and those omitted (*n* = 1489). Participants not included were younger, *F*(1, 2244) = 1.19, *p* = < .001; more likely to be Black, *F*(1, 2244) = 1.12, *p* = .004, or Asian, *F*(1, 2244) = 14.71, *p* = < .001; had fewer household members, *F*(1, 2244) = 72.49, *p* = < .001; decreased private garden access, *F*(1, 1312) = 9.98, *p* = .002, a higher proportion of inaccessible garden space, *F*(1, 1312) = 6.03, *p* = .014; and lower engagement in MVPA in April 2020, *F*(1, 2088) = 33.61, *p* = < .001.

Table 2 shows the final growth curve model for MVPA. Limited access to a private garden or other outdoor space, lower income, female gender, living with a partner, and being of Mixed or Asian ethnicity (with White as the reference group) were associated with lower weekly MVPA at the intercept (April 2020). The remaining ethnic groups (black and other), garden categories (shared and balcony, rooftop garden or terrace), cigarette use, total household composition, mental health condition, and physical health condition were non-significant and omited from the final model for the sake of parsimony [13].

**Table 2:** Growth curve model predicting MVPA trajectory.

Measure	Coef.	SE
**For Intercept**		
Intercept	2.61***^a^	.37
Gender	.42	.27
Mixed Ethnicity	-1.07* ^c^	.51
Asian	-1.24***^a^	.32
Black	-1.62* ^c^	.81
Other	-2.24	2.10
Household Income	.08** ^b^	.02
Living with Partner	-.44* ^c^	.20
Private garden	.71* ^c^	.33
Shared Garden	.68	.47
Balcony, Rooftop Garden or Terrace	.87	.54
Other Outdoor space	1.14* ^c^	.52
**For Linear Slope**		
Intercept	-.09	.08
Gender x Linear Time	.58***^a^	.14
**For Quadratic Slope**		
Intercept	-.00	.00
Gender x Quadratic Time	-.06***^a^	.02
**Residual Variance**		
For Intercept	4.97***^a^	.46

^a^***p < .001

^b^** p < .01

^c^* p < .05.

**Fig 1:**
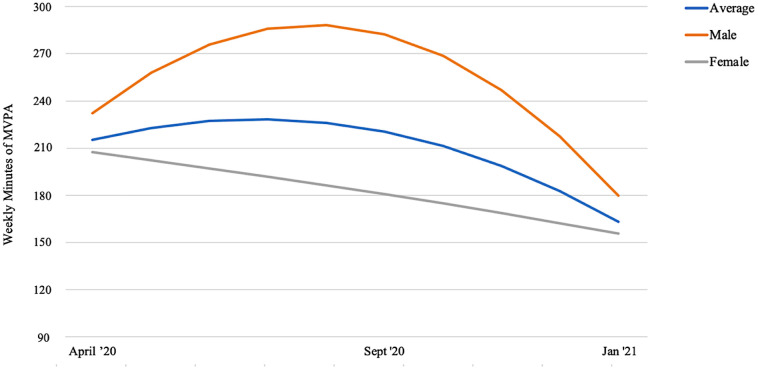
Growth curves showing the trajectories of MVPA for males, females and the sample average from April 2020 to January 2021.

The linear and quadratic slopes were non-significant. However, a gender x linear time interaction revealed a positive linear slope whereas gender x quadratic time interaction revealed a negative quadratic slope (see Table 2). As presented in Fig 1, male participants increased their weekly MVPA from April 2020 until August 2020, followed by a sharp decline. By January 2021, males’ MVPA reached its lowest level. Females presented a significantly lower level of MVPA compared to males throughout and a steady decrease from April 2020 until January 2021. The linear and quadratic slopes were not found to differ according to any of the other covariates.

## Discussion

Utilising the Understanding Society COVID-19 Survey, the present study examined (1) the trajectory of MVPA in young adults and (2) the mean level and rate of change of MVPA according to key socio-demographic and health-related covariates. Although it was expected that MVPA outcomes would align with restrictive measures, declining during the tightening of restrictions and improving upon the easing of restrictions, GCM revealed no significant variation in average weekly MVPA between April 2020 and January 2021. Partial support was observed for the covariates hypothesized to predict worse MVPA outcomes. Female gender, Asian and Mixed ethnicity, lower income, and limited access to a private garden or other outdoor space were associated with lower weekly MVPA in April 2020.

Despite the varying enforcement of restrictions, the average MVPA remained stable and higher than the recommended 75-150-minute baseline between April 2020 and January 2021 for this sample, contrasting prior research [3, 4]. One explanation for the apparent increase in MVPA for young adults is that they became motivated to exercise to compensate for the aversive effects of lockdown periods on weight gain, eating behaviour, and impaired mood [16]. This stance aligns with pre-pandemic literature demonstrating strong associations between intrinsic motives such as the perceived benefits of exercise and favourable exercise outcomes during early adulthood [17].

Supporting and extending prior research, the current study identified that females presented significantly lower MVPA with a persistent rate of decline throughout the pandemic [5, 8]. By contrast, male participants increased their weekly MVPA engagement from April 2020 until August 2020, followed by a sharp rate of decline until January 2021. Work-related changes during the pandemic primarily represented within female populations may account for this gender inequality. Specifically, women under the age of 29 occupy approximately 70% of the global health and social care workforce, thus were more prone to overworking and chronic fatigue as the strain on UK medical services increased during the pandemic [18, 19]. Men–representing the minority of the global health and social care workforce—may have perceived a greater physical opportunity for exercise (e.g., sufficient time) which has been evidenced to promote physical activity in UK adults during the early pandemic [20]. Alternatively, it is possible that female participants perceived greater barriers to physical exercise due to increased childcare demands following the closure of schools and nurseries [18]. However, the present study did not include covariates for employment nor changes to childcare provision in the model, which may be examined in future longitudinal research to corroborate these possible explanations.

In support of previous research [4, 6], this study found that lower income young adults reported lower MVPA. Since economically disadvantaged UK individuals are more likely to live in densely-populated cities with limited access to personal or public green space to exercise [21], they may have been disproportionately affected by the recurrent closure of gyms and the banning of structured team sports. Ethnic minority groups (Mixed, Asian, and Black) were similarly associated with poorer MVPA outcomes in the current study, aligning with previous literature [7]. Given the stratification of income class by ethnicity [22], this finding may also be understood via impoverished accessibility to personal and public outdoor space. The intersectionality between these factors and MVPA provides an important avenue for further research and emphasises the importance of directing resources toward improving MVPA engagement within disadvantaged communities, such as investing in community exercise spaces and increasing accessibility to active forms of transport among other strategies.

In an extension of previous research showing positive associations between MVPA and access to outdoor space during the early months of the pandemic [8, 23], the findings suggest that the inaccessibility of private gardens or other outdoor spaces was associated with poorer MVPA outcomes in April 2020. Due to concerns about COVID-19 infection, shared gardens may have been avoided rendering the facilitation, or conversely prevention, of exercise within these spaces [24]. Reasons for the lack of significance for the balcony, rooftop garden or terrace category are less clear but could reflect the relative lack of exercise space offered by these outlets.

Living with a partner was unexpectedly associated with worse MVPA outcomes in April 2020. While exercise has been cited as a means of coping with pandemic-related stress, research suggests young adults derived significant support from romantic partners over the first lockdown [25], which may have offset the importance of exercise as a means of coping.

Inconsistent with previous research [4, 9, 10], no associations were found between MVPA and cigarette use, mental health condition or physical health condition. However, this could be due to methodological constraints of the final analytic sample. For instance, only 7.9% of respondents provided valid smoking data.

### Further strengths and limitations

Several limitations should be considered prior to the practical application of our findings. Firstly, covariate data that was likely to change during the pandemic, such as income and household composition, were obtained from the first or second waves of data collection. Thus, the dynamic moderation of MVPA outcomes by these predictors was not captured [13]. Moreover, several covariates which may be associated with MVPA outcomes during COVID-19, such as employment status and the provision of childcare [4, 10, 18], were not included in analyses. Further longitudinal research employing a wider range of covariates is warranted to corroborate the findings of the present study and to explore other potential predictors of MVPA during the pandemic.

Secondly, males and BAME groups were under-represented in the sample. Therefore, the full extent of MVPA variation within these demographic groups may have been under-represented. Furthermore, a significant number of participants (*n* = 1489) were excluded from the final analytic sample due to the requirements of GCM. Comparative analyses between these groups revealed several differences in participant characteristics, which, if represented, may have altered the results. Despite the data being drawn from a nationally representative data set, these limitations highlight biases in the present sample and emphasize the importance of further longitudinal research with larger and more diverse samples.

Lastly, elements of the questionnaire content and distribution limited the present findings. For instance, MVPA outcomes were collected via self-report. As participants are evidenced to answer questions relating to personal health and well-being in a socially desirable manner [26], MVPA may have been over-reported by the current sample. That said, the IPAQ research committee’s recommended cut-off of 16 hours or more for totalled weekly exercise estimates was employed for our MVPA data to mitigate over-reporting [14]. Additional research efforts may consider corroborating the present findings via more objective means, such as exercise data tracking technology.

### Conclusions

Despite these limitations, the current study provides novel insight into the trajectory and associated risk factors of MVPA for young UK adults during COVID-19. The findings suggest that further efforts should be made to incorporate physical activity promotion initiatives, such as the “Join the Movement” and “This Girl Can” campaigns set up by Sport England within academic and professional workplaces. Resources can also be directed towards more proximate modes of promoting physical activity, such as increasing accessibility to exercise outlets within economically deprived communities, investing in activity-friendly routes for commuting or leisure, or hosting subsidized community sport classes among other strategies. Overall, the findings contribute to the growing body of COVID-19 literature, providing direction for further research and aiding the development of immediate and future interventions.
